# Supplementary material for: Are carbon emissions trading and green financial instruments synergistic? -Comprehensive quantitative research based on content analysis
Source: PLoS One. 2024 Mar 7;19(3):e0298601. doi: 10.1371/journal.pone.0298601 (PMC10919601; doi:10.1371/journal.pone.0298601)
Supplement: S1 Data — (ZIP) [file pone.0298601.s001.zip › Data/地级市Z_F工作报告.zip!/║╙▒▒/│╨╡┬╩╨/└┤╫╘╣1⁄2╓┌║┼í╛┬φ┐╦╩2╛▌═°í┐_2016.docx]

各位代表：

从今年开始，我们进入第十三个五年规划时期，全市经济社会发展已掀开新的篇章。根据党的十八届五中全会、省委八届十二次全会和市委十三届八次全会精神，市政府编制了《承德市国民经济和社会发展第十三个五年规划纲要(草案)》。现在，我代表市人民政府向大会作工作报告，请各位代表连同《纲要(草案)》一并审议，并请政协委员和列席会议的同志提出意见。

一、“十二五”时期经济社会发展回顾

“十二五”时期是我市积极应对严峻复杂形势、经济持续健康发展的五年，是抢抓重大历史机遇、加快发展方式转变的五年，是经济总量迅速扩大、经济实力大幅提升的五年，是社会事业全面进步、人民生活不断改善的五年。特别是党的十八大以来，在中共承德市委领导下，全市上下深入学习贯彻习近平总书记系列重要讲话精神，全面落实党中央、国务院和省委、省政府的重大决策部署，主动适应经济发展新常态，坚持稳中求进总基调，克服困难，砥砺奋进，朝着全面建成小康社会的目标迈出坚实步伐。

五年来，我们坚持把发展作为第一要务，全力以赴稳增长、扩总量，综合经济实力稳步提升。全市生产总值由2010年的889亿元增加到2015年的 1358.6亿元、年均增长9%左右;人均生产总值由2.57万元增加到3.8万元、年均增长8.6%;全部财政收入由114亿元增加到163.5亿元、年均增长7.5%，公共财政预算收入由54.8亿元增加到97.4亿元、年均增长12%;规模以上工业增加值达到468亿元、年均增长10.7%;全社会固定资产投资1532亿元、年均增长15.3%;社会消费品零售总额489亿元、年均增长13.9%。经济发展规模和水平跃上新台阶。

五年来，我们坚持把产业转型升级作为重中之重，毫不放松提质量、增效益，结构调整力度不断加大。三次产业结构得到优化，由“十一五”末的 15.7：51：33.3调整为17.3:46.9:35.8。大力化解过剩产能，压减炼钢产能240万吨，淘汰水泥产能401万吨，整合关停矿山企业 116家，“两黑”产业占规模以上工业增加值比重由“十一五”末的72.3%下降到66.3%。大力实施工业技改攻坚，累计投资1858亿元，完成亿元以上技改项目658个，净增规模以上企业117家。清洁能源电力装机达到332万千瓦,被列为全国新能源示范市。高新技术产业增加值达到35.8亿元，年均增长13%。大力提升服务业，旅游总收入是“十一五”的2.6倍，电子商务交易和网络零售额达到390亿元，服务业对经济增长贡献率逐步扩大。大力发展现代农业，粮食总产量达到121.2万吨，市级及以上农业产业化龙头企业357家，农业产业化经营率67.3%。新增国家和省级现代农业示范园区6个。围场成功争列国家级现代农业示范区，宽城、滦平列为全国电子商务进农村综合示范县360小说。

五年来，我们坚持把生态作为核心优势，坚持不懈治污染、减排放，生态环境质量持续改善。坚决向大气污染宣战，实施节能改造和污染减排项目1063个，取缔燃煤锅炉776台，治理矿山867个，淘汰黄标车45897辆，在全省率先全部淘汰实心粘土砖，推广使用清洁炉具3.8万台，超额完成节能减排目标，空气质量二级以上天数始终保持在240天以上。加大生态治理与修复力度，新增造林绿化面积405万亩，森林覆盖率达到56.7%;治理水土流失2708平方公里;出境断面和饮用水水源地水质达标率持续保持在100%，水环境质量保持全省第一位。积极探索建立生态补偿和环境保护机制，在全国率先开展跨区域碳汇和碳排放权交易试点。成功争列国家生态文明先行示范区和国家江河湖泊综合整治试点，生态环境质量保持华北最优，生态引力日益彰显。

五年来，我们坚持把基础设施建设作为重要突破，持之以恒打基础、利长远，制约发展的短板显著改善。把破解交通制约作为重中之重，整合资源，合力攻坚。承赤、承秦、承围、承张四条高速建成通车，“一环八射”高速路网框架初步形成，高速公路里程达到757公里，比“十一五”末增加473公里;国省干道改造步伐加快，二级及以上等级公路达到3049公里，比“十一五”末增加1138公里;新建农村公路6400公里，行政村全部实现村村通公路。京沈客专全面开工，张唐铁路竣工，铁路运营里程达到796公里、比“十一五”末增加215公里。承德机场主体工程基本完成。实施重点水利工程157项，总库容达到4.1 亿立方米，双峰寺水库一期大坝主体完工，丰宁抽水蓄能电站开工建设。交通、水利、电力、通讯等基础设施得到全面提升政府工作报告。

五年来，我们坚持把新型城镇化作为关键支撑，坚定不移抓城市、带农村，统筹城乡步伐明显加快。城市规划、建设和管理水平不断提升，“中疏”战略取得阶段性成果，实施水电路气等市政基础设施和文教卫体等公共服务项目837个，建成区面积从97平方公里扩大到112平方公里。县城容貌和承载功能加快提升，建成区总面积增加到79平方公里，宽城“产城教”一体化模式在全省推广，国家级和省级园林县城分别达到3个和8个，省级以上文明县城、卫生县城分别达到3个和 7个。特色小城镇加快发展，建制镇达到90个，有20个镇晋升为全国重点镇。农村人居环境不断改善，改造提升美丽乡村省级重点村665个。出台户籍改革相关政策，有序放开城镇落户限制，城镇化率达到46%，比“十一五”末提高6.6个百分点。以城带乡、城乡一体的发展格局逐步形成。

五年来，我们坚持把深化改革作为根本动力，千方百计破瓶颈、解难题，重点领域改革全面推进。以前所未有的力度推进简政放权，全部取消非行政许可审批事项，市级取消审批事项93项，向县区下放行政权力291项。市本级实施重点项目全程分段代办制，重大项目审批时限缩减至30个工作日以内。市县全部建立政务服务中心，“三级平台”、“两个代办”实现全覆盖，乡镇便民服务中心覆盖率达到98%。商事登记制度改革实现突破，实施“三证合一”、“先照后证”，市场主体比“十一五”末增加6.6万户。大力推进金融改革创新，新增外埠银行6家，新增上市挂牌企业13家，整合近百亿国有资产组建国控集团。农村综合改革稳步推进，供销社综合改革被列为全国农村合作金融体系建设试点。兴隆被列为全国农田水利产权制度改革和创新运行管护机制试点。隆化、丰宁被列入全国国有林场改革试点。

五年来，我们坚持把扩大开放作为突出任务，大力推动引进来、走出去，协同发展取得可喜成果。积极实施京津冀协同发展重大国家战略，87项重大事项和项目纳入国家和省及京津战略规划层面，精准对接国家部委、行业协会、科研院所、央企京企，实施合作项目124个，完成投资350亿元。携手中关村打造中关村·承德协同创新共同体示范区，绿色大数据、电子信息、文化创意、节能环保等一批新兴产业项目签约落地，承德县被评为“省级服务外包呼叫中心基地”。产业承载空间不断扩大，承德高新区晋升为国家级高新区，省级以上开发区达到13家。丰宁、滦平、兴隆三县开通北京城际公交。多渠道、全方位开展对外招商，实际利用外资5.4亿美元，是“十一五”的2倍，外贸进出口总额是“十一五”的1.2倍，累计引进市外资金2230亿元。承德海关开关运行，隆化保税仓库建成投用，平泉创建成为国家级出口食品农产品质量安全示范区。对外开放的广度深度进一步拓展。

五年来，我们坚持把改善民生作为根本目标，尽心竭力保基本、兜底线，人民群众的获得感进一步增强。持续加大民生投入力度，财政民生投入累计达到949亿元，是“十一五”的3.1倍，占全部财政支出的76.1%。城乡居民人均可支配收入稳步增长，分别达到22885元、7923元，是2010年的1.7倍和1.8倍。扎实推进扶贫攻坚，66.5万贫困群众实现稳定脱贫，318个贫困村脱贫出列。新增城镇就业22.2万人次，农村劳动力转移就业27.8万人次，城镇登记失业率控制在4.5%以内。教育事业长足发展，国家级义务教育均衡发展县区达到6个，被国务院表彰为全国“两基”工作先进地区。县级公立医院综合改革实现全覆盖，县乡卫生服务一体化管理走在全省前列。人口计生工作保持全省领先。社会保障体系不断完善，城乡低保标准高于全国平均水平，医疗保险实现市域内异地就医即时结算，企业退休人员基本养老金由1363元提高到1997元，城乡居民社会养老保险制度实现全覆盖。建成保障性住房6.8万套，改造农村危房10.2万户。解决了141.4万农村人口饮水安全问题。公共文化服务体系不断完善，国家文献战略储备基地、中关村互联网文化创意产业园等一批重大文化产业项目落地，《鼎盛王朝·康熙大典》实景演出成为靓丽的文化名片，电视剧《打狗棍》在全国产生广泛影响。营子区被评为全省公共文化服务示范区。社会管理创新深入推进，双桥区、双滦区被确定为全国和谐社区建设示范城区、全国社区治理和服务创新试验区。双拥共建结出新硕果，成功实现全国双拥模范城“八连冠”。法治承德、平安承德建设扎实推进，安全生产形势保持平稳，社会大局和谐稳定。民族宗教、外事侨务、人民防空、史志档案、统计调查、气象地震、防灾减灾、对口援疆、邮政通信、妇女儿童、老龄、残疾人等工作取得新成绩。

各位代表!刚刚过去的2015年，面对经济下行的困难形势，我们坚持解放思想、抢抓机遇、奋发作为、协同发展，进一步巩固深化党的群众路线教育实践活动成果，扎实开展“三严三实”专题教育和解放思想大讨论，树正气、讲团结、聚合力，在困难中坚定信心，在挑战中开拓进取，全力以赴上项目，深化改革破瓶颈，精准对接促协同，一批稳增长的政策措施取得实效，一批重大事项上升到国家层面，一批新兴产业起步发展，一批民生实事得到有效落实。全市生产总值增长 5.5%，规模以上工业增加值增长4%，固定资产投资增长7.8%，社会消费品零售额增长9.4%，经济运行总体保持在合理区间，为“十三五”发展奠定了坚实基础。我们自觉接受人大、政协监督，及时办理人大代表建议和政协委员提案，办结率均达到100%。

各位代表!过去五年取得的成绩来之不易，积累的经验弥足珍贵，为我们全面做好“十三五”各项工作，推进全市经济社会又好又快发展提供了许多有益的启示：必须始终坚持发展第一要务，直面挑战，坚定信心，攻坚克难，发奋图强，尽快改变承德经济欠发达的现状;必须始终坚持转型升级主攻方向，树立全新发展理念，摆脱传统路径依赖，坚定不移走“差异化、特色化、增量调整”发展之路，推动经济提质增效;必须始终坚持生态优先不动摇，既要“金山银山”，更要“绿水青山”，坚守生态与发展“两条底线”，进一步放大生态承德的品牌效应;必须始终坚持改革创新不停步，坚决破除制约和阻碍发展的体制机制障碍，不断激发加快发展的内生动力，进一步释放企业和社会创新的活力;必须始终坚持牢牢抓住协同发展的机遇和大势，全方位扩大开放，真正把机遇优势转化为发展优势;必须始终坚持把改善人民生活、增进人民福祉放在重中之重的位置，着力解决人民群众最关心最现实的利益问题，使发展成果更多更公平地惠及全市人民;必须始终坚持践行 “三严三实”，在改进作风上下功夫，简政放权，优化环境，不断激发市场的活力和社会创造力，汇聚正能量，推动大发展。

各位代表!五年来，我市经济社会发展取得的成就，得益于党中央、国务院的亲切关怀，得益于省委、省政府和市委的正确领导，得益于市人大、市政协和社会各界的大力支持，得益于全市广大干部群众的团结协作、奋力拼搏。在此，我代表承德市人民政府，向全市380万人民，向人大代表、政协委员，向各民主党派、工商联、无党派人士和人民团体，向驻承人民解放军、武警部队官兵和政法干警，向一直以来关心和支持承德经济社会发展的国内外各界朋友，表示崇高的敬意和衷心的感谢!二、“十三五”时期经济社会发展的主要任务

“十三五”是我们加快发展、绿色崛起的机遇期，是我们结构调整、转型升级的关键期，是我们脱贫摘帽、全面小康的决胜期。摆在我们面前的，既有诸多有利因素，也有巨大的困难和挑战。

我们要看到，当前承德的经济社会发展正处在一个十分特殊的历史时期，我们面临的经济下行压力前所未有，经历的转型阵痛前所未有，承受的困难挑战前所未有。特别是刚刚过去的2015年，受外部经济环境影响，加之承德总量小、底子薄、基础脆弱，经济欠发达的基本市情尚未得到根本性改变，经济增长进入“低谷”，增速为本世纪以来最低，生产总值、财政收入、规模以上工业增加值等几项主要经济指标未能完成年初计划，一些长期累积的矛盾和问题集中爆发和显现，主要体现在：一是增长方式粗放。“两黑”产业占规模以上工业比重过高，产业结构性矛盾突出，生产链条短，产业产品层级低。随着“两黑”产业产品价格下降，效益下滑，原有增长动力大幅衰减，新的增长动力尚未形成，保增长、调结构、转方式、防风险的任务迫在眉睫;二是基础支撑能力弱。中心城市和县城辐射带动能力不强，园区发展规模小、水平低，交通、水利、电力等基础设施欠账多，教育、科技、文化、卫生等公共服务滞后，难以支撑经济社会持续快速健康发展;三是对外开放水平低。一些干部思想僵化保守，思想观念、思维方式与新常态、新要求、新任务不相适应，对外开放的层次、水平、程度不高，制约发展的体制机制障碍亟待破解;四是脱贫攻坚任务繁重。全市八县均属燕山—太行山特困片区及环首都贫困带，尚有906个贫困村，50.07万贫困人口，贫困面较大，贫困程度深，补短板、兜底线，实现脱贫摘帽、全面小康的任务十分紧迫;五是经济发展环境欠优。政府职能转变还不到位，一些地方和部门缺乏改革精神、法治精神、担当精神，尊商、亲商、重商的氛围不浓，不作为、慢作为、乱作为的问题比较突出，消极腐败现象依然存在，人民群众对政府的效率、作风还不满意，政府自身建设亟待加强，等等。对这些问题，我们必须实事求是、勇于面对，采取务实管用举措，逐个加以破解。

与此同时，我们更要看到，当前承德的发展面临着诸多重大历史性机遇。我国经济发展步入新常态，仍处于可以大有作为的重要战略机遇期，为承德发展提供的良好外部环境十分难得;国家深入实施京津冀协同发展、创新驱动和脱贫攻坚等一系列重大战略部署，为我们培育新的发展动力、赢得更多支持提供的机遇千载难逢;中央提出加快推进供给侧结构性改革，为我们产业转型升级、企业加快调整、资源要素配置带来的一系列重大利好前所未有。从承德自身来讲，越是经济发展困难的时候，越是转型创新的难得机遇，特别是全市上下政通人和，生态引力日益彰显、基础设施逐步改善、项目支撑能力不断增强，承德的比较优势和发展后劲必将得到充分释放。我们一定要提振士气、振奋精神，敢于直面困难和压力，善于应对矛盾和问题，增强信心，坚定决心，主动适应和引领新常态，把思想统一到发展上、把心思集中到发展上、把力量凝聚到发展上，奋力开启承德又好又快发展的新征程!

“十三五”时期我市经济社会发展的指导思想是：全面落实党的十八大和十八届三中、四中、五中全会及省委八届十二次全会精神，深入学习贯彻习近平总书记系列重要讲话精神，遵循“五位一体”总体布局和“四个全面”战略布局，高举发展、团结、奋斗旗帜，按照市委十三届八次全会部署，树立绿色、创新、开放、转型、协调、共享的发展理念，坚持守底线、快转型、促协同、奔小康的主基调，围绕“京津冀水源涵养功能区、国家绿色发展先行区、环首都扶贫攻坚示范区、国际旅游城市”发展定位，大力推进新型工业化、信息化、城镇化和农业现代化，大力推进转型升级，大力推进脱贫攻坚，大力推进“五城联创”，加快建设生态强市、魅力承德，确保如期实现全面建成小康社会目标，为谱写中华民族伟大复兴中国梦的承德篇章奠定更加坚实的基础。

“十三五”时期我市经济社会发展的总体目标是：“一个领先”、“两个翻番”、“三个提升”。即：生态环境质量在京津冀保持领先;生产总值比2010年翻一番以上、年均增速7.5%左右，城乡居民人均可支配收入比2010年翻一番以上;区域综合经济实力、城镇化水平和城乡居民幸福指数比“十二五”大幅提升，全面建成小康社会。我们坚信，只要全市上下团结一心，攻坚克难，埋头苦干，一个经济更加繁荣、社会更加和谐、生态更加优良、人民更加幸福的承德必将呈现在塞北大地!

今后五年，我们要重点完成好以下六大任务：

(一)坚定不移推动绿色发展，着力在生态文明建设上达到新境界。生态优势是承德的第一优势，是承德可持续发展的核心竞争力。我们必须牢固树立“绿水青山” 就是“金山银山”的理念，加快把生态优势转化为发展优势，打造生态最美的承德、环境最优的承德、品牌最亮的承德。涵养调蓄水源，让河水更清。坚持“以水定城、以水定产、以水定发展”，围绕“六水共治”，抓好21世纪首水、滦河干流独流入海等重大工程建设，实施双峰寺水库等控制性蓄水及水网工程。加快全流域水环境综合治理和生态修复，严格水资源管理，严控地下水开采，培育涉水高端产业。到2020年，水资源控制率达到20%以上，新增蓄水4亿立方米，湿地面积保持在6万公顷以上，水土流失治理率达到50%以上。筑牢生态屏障，让山川更绿。推进京津风沙源治理、京冀津冀水源林、再造“三个塞罕坝”、再造“一个坝上草原”等重点生态工程。开展“强基护绿”工程。到2020年，森林覆盖率达到并稳定在60%以上，完成退化草地治理58万亩。全市经济林总规模达到 1000万亩，产值1000亿元以上。提升空气质量，让天空更蓝。用铁的手腕、铁的措施，坚决打好减煤、治企、控车、抑尘、多污染物治理大气污染防治“五大攻坚战”。大力推广应用风能、光能、水能、天然气等清洁能源，在农村和城郊村推广使用洁净型煤、木煤等环保燃料和高效洁净炉具，确保空气质量二级以上天数占比达到75%以上。强化土地整治，让大地更洁。加强土地、矿产等资源的节约循环利用，实施农业面源污染治理行动计划，严格控制和治理土壤污染，实现化肥农药零增长。开展国家绿色矿业发展示范区创建，启动实施矿山综合整治“四个一批”工程，利用3—5年时间，完成损毁山体治理60平方公里，压减矿业权 700个，引导矿山企业转产转型400家以上，淘汰取缔300家左右。加强生态文明机制建设，完善自然资源负债表编制方式方法，探索用能权、用水权、排污权、碳排放权等生态产品交易方式，打造生态产品交易平台，加大合同能源管理和环境污染第三方治理推广应用力度，推动建立滦河、潮河流域跨界水环境横向生态补偿机制。坚定不移走绿色发展之路，努力让承德青山常在、绿水长流、蓝天永驻。

(二)坚定不移推动创新发展，着力在培育发展动能上实现新突破。改革创新是加快发展的动力之源。我们必须把发展的基点放在创新上，大力实施创新驱动战略，加快培育发展的新动能。加快体制机制创新释放新红利。加大供给侧结构性改革力度，提高全要素生产率，扩大有效供给。深入推进简政放权，加强监管，优化服务，提高行政审批效能。深化农村金融、土地承包经营权、农村集体经营性资产等领域改革，盘活农村“沉睡”资源资产。深化投融资改革，完善国控集团等市场化融资平台运作机制。推动企业上市融资，扩大直接融资比重，力争到2020年引进金融保险机构3—5家，多层次资本市场上市企业达到40家以上。大力支持非公有制经济发展，搭建企业家成长平台，加大企业家培训力度，培育一支优秀的企业家队伍，民营经济占比达到75%以上。加快推动国有企业改革，利用2—3年时间彻底解决市属破产国有企业改革遗留问题。加快科技创新打造新优势。以建设中关村·承德协同创新共同体示范区为突破，以实施“十大科技工程”为抓手，完善协同创新体系，推进科技成果转化，与京津共建一批高水平工程技术研究中心和产业技术研究院，引进培养一批行业领军人才和创新创业团队，培育壮大一批高新技术企业，科技型中小企业达到1200家、高新技术企业达到50家、科技“小巨人”企业100家、各类科技研发和公共服务机构60家，力争建成国家级工程技术研究中心1家，每万人发明专利拥有量年均增长17%以上。加快产业创新拓展新领域。深入实施“互联网+”行动计划，推动互联网与生态环保、文化旅游、装备制造、现代农业、现代物流的深度融合，扩大新产品和服务供给，以供给创新释放消费潜力，开辟区域发展、产业发展、网络经济新空间，推动创新成果变成实实在在的产业活动，力争规模以上高新技术产业增加值年均增长12%以上。我们一定努力营造大众创业、万众创新的浓厚氛围，让创新创业在承德大地热流涌动、竞相迸发。

(三)坚定不移推动开放发展，着力在对内对外开放上迈出新步伐。置身京津冀协同发展大背景，我们必须靠开放拓展空间，靠开放引领发展，着力增强经济发展的新活力。立足京津冀，推动协同发展。瞄准“三区一城”功能定位，围绕列入中央和京津规划战略层面的重大事项，加快推进交通互联、产业互接、能源互补、生态共建、旅游互动，力争国家绿色发展先行区建设取得明显成效。坚持“五合一”精准对接模式，积极承接非首都功能疏解和产业转移，在医疗教育等领域开展多种形式合作，推动电子信息、大数据、文化创意、健康养生、高端旅游产业加快发展，努力形成协同发展、互利共赢的新格局。面向海内外，实现借力发展。借力国家 “一带一路”战略，推进钢铁、水泥等优势产能走出去，全力拓展中亚、欧洲和俄罗斯市场。借鉴国内自由贸易试验区经验，支持有条件的园区设立保税监管场所，形成对外开放功能区。发挥好承德海关作用，加快电子口岸、国际贸易单一窗口建设，提升贸易能力。依托香港园区招商办、欧洲招商展示中心等平台，加大攻关对接力度，办好系列对外开放活动，引进一批重大外资项目。力争利用外资8亿美元以上，外贸出口24亿美元。做强开发区，加快集群发展。改革优化开发区管理体制机制，以市场方式完善基础设施建设，以优质服务推动项目招商建设。加快构建以承德高新区为核心，以各县经济开发区及特色园区为支撑的产业发展平台，高起点打造大数据产业城、高端装备制造产业城、通用航空城，提高人才、科技等先进要素的聚集度。到2020年，全市园区主营业务收入超1000亿元的1家、超 500亿元的2家、超100亿元的8家，为实施“引进来”、“走出去”双向开放战略打造平台、创造条件。

(四)坚定不移推动转型发展，着力在产业结构调整上实现新跨越。坚持“差异化、特色化、增量调整”思路，强化问题导向，倒逼转型升级，以十大绿色产业为抓手，打造和培育新的经济增长点。推动传统产业向中高端迈进。加快实施工业强基战略，改造提升黑色金属冶炼和压延业，增创发展新优势。推动汽车零部件、输送装备、智能仪器仪表等产业向高端化、智能化、链式化方向发展，引进通用飞机及航空零部件、新能源汽车、混合动力系统等高端装备制造业，逐步培育工业机器人、新型传感器等制造产业体系。推进建材产业向新型绿色低碳发展，打造京津冀新型建材及建筑产业化基地。引进战略投资者，打响“京津水源·皇家山泉”品牌，建设中国高锶矿泉城，推动天然山泉水产业做大做强。力争到2020年，先进装备制造业主营业务收入达到800亿元，新型建材产值达到500亿元以上，天然山泉水销售收入达到100亿元以上。培育壮大战略性新兴产业。围绕打造国家级清洁能源产业基地，科学布局，有序发展水电、风电、光伏发电及核电，建成投产百万千瓦风电二期工程，启动实施百万千瓦风电三期工程，清洁能源电力装机达到1200万千瓦，实现产值310亿元。围绕建设国家级绿色钒钛产业基地，大力提升提钒、提钛技术，开发钒钛高端用钢和钒钛制品、钒钛新材料，推进全钒液流储能电池产业化、粉末冶金产业化，力争钒钛新材料产值达到1000亿元。围绕打造京津冀节能环保产业园，强化生态智慧、技术引领理念，深化与中关村智慧环境产业联盟、中节能、博天环境等知名企业合作，推进环境污染第三方治理，节能环保产业销售收入达到1000亿元。突出发展现代服务业。围绕建设国际化休闲旅游目的地和中国健康养生基地，依托大企业大集团，推动旅游与文化体育、休闲康养、现代农业、工业及相关产业的融合发展。围绕“两带、三环、五大精品旅游板块”，着力培育冰雪温泉、狩猎马术、低空飞行、房车露营等新产品、新业态，打造全域旅游目的地县区，打造一批高端核心景区和特色旅游村镇，提高旅游业附加值，提升国际旅游城市品质和影响力。大力培育文博印刷、影视娱乐、动漫创意等新兴业态，全力把文化旅游业打造成现代服务业的主导产业和国民经济战略支柱产业，旅游总收入突破1000亿元，旅游业增加值占全市生产总值的比重达到15%以上。围绕建设“国家绿色大数据中心”，依托中关村大数据产业联盟，以中国电子信息生态科技城及承德高新区大数据产业园为平台，加快打造“六大基地”，争创全国服务外包示范城市，建设“京津冀大数据走廊”重点支撑城市，大数据产值达到500亿元以上，带动相关产业规模1000亿元。围绕建设连接京津冀辽蒙的重要物流枢纽，着力打造交通枢纽型、生产基地型、商贸集散型物流聚集区，全力发展第三方物流，大力发展电子商务，建成国家电子商务示范城市，物流业实现增加值240亿元。加快发展现代农业。依托特殊的气候条件以及“京津水源、皇家农庄、道地药材”等金字招牌，强龙头、扩基地、建园区、育品牌，做强蔬菜、食用菌、林果、畜牧、中药材五大主导产业，做优水产、马铃薯、专用玉米、杂粮等区域特色产业，做大休闲农业、体验农业、智慧农业、功能农业等新型业态，叫响“京津水源地·承德生态农产品”区域品牌。发展壮大农产品加工龙头企业30家以上，培育“颈复康”、“神栗”等一批知名企业，打造中国驰名商标和河北省著名商标20个以上，绿色食品及生物医药产业实现产值1000亿元以上，初步建成京津绿色有机食品直供地、中国北方最大的食用菌生产销售集散地和河北省燕山中药材经济核心示范区。

(五)坚定不移推动协调发展，着力在区域均衡发展上取得新成效。协调是实现又好又快发展的内在要求。我们必须在协调推进中增创发展优势，在统筹发展中补齐发展短板，在加强薄弱领域中增强发展后劲。推进区域协调发展。深度融入京津冀区域协同发展大局，按照“两区两带”空间布局，以中关村协同创新共同体示范区为依托，支持双桥、双滦、高新区及承德县建设中部承接非首都功能疏解核心区，重点吸引京津高端产业和教育文化医疗等公共资源转移;支持围场、丰宁和隆化建设北部生态涵养支撑区，重点推进生态建设产业化、产业发展生态化;支持滦平、兴隆、营子及丰宁中南部着力塑造独具魅力的城市品牌，建设西部环京津产业承载隆起带，发展服务京津的高端旅游、健康养生等产业，承接北京产业外溢，建设上下游链接配套的产业基地;支持平泉、宽城和围场及隆化中南部建设东部出海通道产业聚集隆起带，重点打造连通辽蒙与沿海、港口与腹地的经济走廊和绿色产业聚集区。推进城乡一体化发展。以“五城联创”为抓手，突出规划引领，提升城市品位和承载能力，壮大城市经济，构建“一核三带六组团”中心城区形态布局。走小县大县城路子，加快产城教融合发展，打造有历史记忆、地域特征、山清水秀的美丽县城。以国家和省、市级重点镇为主体，加快打造御路文化城镇带、环京津特色城镇带和出海城镇带，培育一批供给侧经济小镇和特色小镇。加快美丽乡村建设，按照“四美五改、六位一体”要求，沿京津、沿景区城区、沿交通主干线，打造9大美丽乡村片区，建设 16个环京津少数民族特色村镇，建成100个省级中心村示范点，完成300个精品村改造提升任务。推进城乡基础设施建设，加快高速公路、铁路建设，高速公路通车里程达到1200公里，铁路通车里程达到1300公里。承德机场开通国内航线10条以上。实施县乡村基础设施“三年攻坚计划”，市域内国道和80% 的省道达到二级以上标准，实现县县通国道，90%的建制镇和60%以上的乡通达二级公路。推进文化、教育、卫生等公共服务向农村延伸，健全村级卫生室、文化站、阅览室，实现垃圾、污水处理村级全覆盖，让农民享受与城市均等化的基本公共服务。到2020年城镇化率达到55%以上。推进物质文明和精神文明协调发展。培育践行社会主义核心价值观，发扬传承中华优秀传统文化，大力弘扬“塞罕坝”精神，完善公共文化服务体系、文化产业体系、文化市场体系，努力形成向上向善、诚信互助的社会新风尚。深入开展群众性精神文明创建活动，加大优秀文艺作品生产扶持力度，打造一批地方标志性特色文化设施，加快推进市级图书馆、群艺馆、博物馆、体育馆建设，建成承德民族团结清文化展览馆，实现村民中心、综合文化服务中心、村史馆(室)和体育健身路径工程全覆盖。

(六)坚定不移推动共享发展，着力在保障改善民生上实现新作为。全面建成小康社会的神圣使命，已经历史地落在我们这一代人肩上，我们必须坚持用脱贫攻坚统揽经济社会发展全局，更加注重民生改善，让人民群众在共建共享中有更多实际获得感。坚决打赢脱贫攻坚硬仗。认真落实习近平总书记“六个精准”“五个一批” “四个切实”的指示精神，围绕实现“两步走”“三确保”“四提升”目标，重点抓好6个跨县域扶贫产业带、9个扶贫产业片区及一批家庭手工业生产扶贫基地，推广“政府+龙头企业+金融机构+合作社+贫困户”一体化扶贫模式，确保到2018年，贫困县全部脱贫摘帽，906个贫困村、50.07万贫困人口实现稳定脱贫，决不让一户困难群众在全面小康的进程中掉队。全力推进社会事业发展。优化基础教育布局，促进义务教育均衡发展，构建现代职业教育体系，注重高等教育内涵发展，教育发展主要指标达到全省先进水平。全面推进公立医院综合改革，完善医疗卫生服务体系。大力发展体育产业，争取国家级射击训练基地、冰雪运动训练基地和电子竞技赛事基地落户承德。加快完善社会保障体系。完善城乡居民养老、医疗和失业、工伤、生育保险制度。城镇居民和新型农村社会养老保险制度全覆盖。城乡居民基本医疗保险参保率达到99%。加快保障性住房、棚户区和城乡危房改造步伐，到“十三五”末，全部完成城市危房改造任务，基本完成中心城区棚户区改造任务。健全养老设施和服务网络，公办示范性养老服务机构实现市县全覆盖，城市街道社区和有条件的行政村建立老年人服务站。加强困难群体救助，发展残疾人事业，建立医疗救助与慈善援助衔接机制和平台，医疗救助重点对象年人均筹资增长达到18%以上。进一步做好各项安全稳定工作，为全市人民创造安定和谐的社会环境。

各位代表!“十三五”的发展大幕已经开启，决战决胜全面建成小康社会的号角已经吹响。只要我们团结一心，担当实干，励精图治，攻坚克难，就一定能够实现全面建成小康社会的奋斗目标，就一定能够铸就承德发展新的辉煌!三、2016年重点工作

今年是“十三五”的开局之年，是京津冀协同发展全面推进的关键之年，也是推进供给侧结构性改革的破冰之年，做好今年各项工作至关重要。我们要按照中央和省各项决策部署，全面贯彻“绿色、创新、开放、转型、协调、共享”的发展理念，适应和引领经济发展新常态，坚持生态保护与经济发展双赢共进，坚持供给侧改革与需求侧改革双向发力，坚持优化存量与扩大增量双轮驱动，坚持借助外力与激发内力双管齐下，加快形成对经济发展稳定而持久的拉动力，确保“十三五”开好局、起好步。今年经济工作的主要目标是：生产总值增长6.5%左右，规模以上工业增加值增长5%，固定资产投资增长10%以上，全部财政收入增长4%，实际利用外资增长5%，出口总额增长5%，社会消费品零售总额增长9%，城乡居民人均可支配收入分别增长9%和10%。重点做好八个方面工作：

(一)强力推进经济稳定增长。坚持把稳增长作为首要任务，加强供给侧与需求侧有效对接，统筹施策，多点支撑，确保经济增长不失速。千方百计上项目、增投资。把项目建设作为重中之重，重点推进“3281”工程，争列省重点项目30个，实施市级重点项目200个，亿元以上项目830个，确定兴市立县、事关全局的重大产业和基础设施项目100个。市级重点启动10亿元以上重大产业项目和30亿元以上重大基础设施项目各10个，县级重点实施3亿元以上重大产业项目和基础设施项目各5个。健全完善项目推进机制，落实市县领导分包“五个一”工作机制，抓好集中开工、观摩拉练和推介招商，组织好百家名企进承德和 “1+4”系列对接活动，推动项目签约落地、开工建设、投产达效，确保产业投资增速在15%以上。千方百计扶企业、增效益。深化“企业帮扶年”活动，落实中央、省和我市减少涉企收费政策，深化政银企互动合作，切实减轻企业负担，提升企业经济效益。推动优势企业整合扩张，通过技改攻坚升级一批、产业扩能发展一批、嫁接改造提升一批、退市进郊搬迁一批，尽最大努力保骨干企业不减产、不停产。重点实施天大钒业、华远自动化等亿元以上技改项目100个。设立市本级 30亿元以上技改专项基金，支持20个重大技改扩能项目建设。各县相应设立技改基金，每县至少实施10个以上重大技改扩能项目。新增规模以上企业40家以上。千方百计扩内需、促消费。顺应和把握消费升级的大趋势，开展改善消费品供给专项行动，开发适合不同群体的多样化、个性化服务需求。抓住国家探索实行夏季2.5天休假模式的机遇，加强旅游基础设施建设，提高旅游产品供给能力。落实职工带薪休假制度，发展夜经济、假日经济，鼓励错峰旅游，有效促进旅游消费。开展“大健康、新医疗”行动，壮大健康、医疗、养老消费。化解房地产库存，推动房地产行业健康发展。畅通“农产品进城”和“消费品下乡”双向流通网络，落实新一轮汽车下乡政策。加快城市商业综合体和社区体验店建设，构建快捷便民生活圈，举办好美食文化节等活动，挖掘消费潜力，增加服务供给，促进经济增长。

(二)强力推进绿色产业体系建设。坚持生态产业化、产业生态化，加快培育壮大十大绿色产业。文化旅游与健康养生产业。重点抓好隆化热河皇家温泉、滦平金山岭国际滑雪度假区、围场御道口等重点旅游休闲度假区建设，全力创建金山岭长城5A级景区，加快推进兴隆山和六里坪5A级景区建设。强力推动中关村互联网文化创意产业园承德园、北京大稿国际艺术园承德园等重大项目建设。加大整体旅游形象品牌的包装推介力度，组织开展国际旅游文化节、冰雪温泉国际旅游节、“禅修之旅”等文化旅游节庆活动，推广“山里寒舍”品牌模式，打造50个乡村旅游精品村。启动智慧旅游城市建设，加快建设游客集散中心。全年接待中外游客 3850万人次、实现旅游收入400亿元，分别增长15%和20%。清洁能源产业。重点抓好丰宁抽水蓄能电站二期、围场光伏发电等重点项目建设，推动长河核电项目争列国家规划。新增清洁能源装机48万千瓦、达到380万千瓦，产业增加值占比提高2个百分点，达到10%以上。钒钛新材料产业。重点抓好钒钛基地一期190万吨冷轧、120万吨提钒转炉工程以及钛能轧钢超薄冷轧带板项目开工建设，确保天大钒业5200吨钛合金一期竣工投产、二期全面开工，力争金科科技1万吨钒氮新材料项目开工。绿色大数据产业。重点抓好中国电子信息生态科技城、河北大数据交易中心、世欣蓝汛等项目，力争上半年开工建设。启动智慧城市、智慧旅游、智慧环保、智慧医疗、智慧农业建设，承德云服务中心挂牌运营。推进与万国、软通动力、清华华唐等企业集团和高校院所合作，实现服务业外包、呼叫中心项目新的突破。节能环保产业。重点抓好致城生物质燃料、国投圣世土壤添加剂研发等18个项目，筹集设立环保产业基金，推进中关村智慧环境产业联盟承德科技园、承德环保仪器设备科研与产业化基地建设。绿色食品及生物医药产业。重点抓好露露迁址扩能、栗源食品工业园、颈复康中药、怡达果品等27个项目，全力推进“百万亩经济林、百万吨食用菌、百万亩中药材花海、百万头优质肉牛、百万只优质肉羊”示范基地建设，建成以热河黄芩为主的中国道地药材价格形成中心和期货交易中心。积极推进国家农业公园项目落户我市。举办好“水源地承德·绿色产品北京行”系列活动。食品医药工业增加值占比提高2个百分点，达到10%以上。天然山泉水产业。重点抓好资源勘查开发利用规划和产业发展规划的编制,年内探明可开发利用矿泉水和山泉水产地30处以上。建设中国(承德)矿泉研发、检验、检测中心，积极引进外埠知名企业开发合作，矿泉水产能达到100万吨以上，销售收入10亿元以上。新型建材产业。重点抓好玄武岩纤维制品、沸石环保新材料、新型绿色自保温建筑材料等10个重点项目建设，河北睿索固废工程技术研究院开工建设，尾矿综合开发利用产值达到160亿元。先进装备制造业。重点抓好平泉希翼风力发电机、承德阀门工业园二期等37个项目建设，加快打造比亚迪新能源客车承德基地，确保亿元以上竣工、开工、技改和前期项目分别达10个以上，力争装备制造业增加值增长7%。现代物流产业。重点抓好承德国际商贸物流园区、平泉华北物流园区、丰宁现代物流园区建设，加快推进高新区农产品冷链物流产业园等59个项目，打造进京农产品冷链物流和物流标准化试点，建设“环首都东北部一小时物流圈”。推进10个电子商务产业园、孵化园建设，建立承德“生态有机农产品电商营销产业联盟”，实现村级电子商务服务全覆盖，新增全国电子商务示范县1-2个。物流业增加值达到120亿元，增长 15%以上。

(三)强力推进京津冀协同发展。坚持借外力、增内力、激活力，巩固扩大协同开放成果。提高跑办对接精准度。立足国家和省协同发展的有关规划及政策，点对点谋划对接，建立对接项目库，确保列入国家和省“三个率先突破”工作方案的20个重大事项和项目如期推进，确保市级领导牵头、有关县区及部门具体跑办的37 个重大事项和60个重点项目取得实实在在成效。提高先进要素聚集度。启动“园区整体提升”三年计划，制定出台加快园区发展的实施意见，设立园区建设专项扶持基金，创新园区的体制机制，提升园区基础配套能力。支持高新区率先突破，重点抓好16个市县两级承接平台，积极推进国别(地区)特色园区建设，实现有序错位发展。加快承德中关村协同创新共同体示范区建设，深化大数据、节能环保、大健康三个重点领域合作。综合运用“周末工程师”等方式，大力引进京津高端人才，实施科技型企业“倍增工程”和“科技小巨人培育计划”，新培育认定科技型中小企业130家、高新技术企业10家、科技小巨人企业13家。提高对外合作开放度。筹备组织好韩国、中东欧等境外系列招商活动，重点推进华润风电增资、承德金源等5个外资项目开工建设，引进市外资金430亿元以上，实际利用外资达到1.7亿美元。制定出台加快发展外贸转型升级的具体措施，支持钢铁、水泥等优势产能走出去，培育新增出口备案企业20家，新增境外投资企业3家以上。

(四)强力推进重点领域改革。以更大的决心和勇气，着力破除制约发展的体制机制障碍。深化行政审批制度改革。深化“放管服”改革，落实重点项目全程代办制，建立行政许可事项等“五张清单”，建设投资项目在线监管平台等“四张网络”，完善商事登记制度改革，清理规范中介机构，推进大众创业、万众创新，市场主体增长达到15%以上，万人拥有市场主体突破500户。深化农村综合改革。基本完成农村土地承包经营权确权登记颁证，土地确权登记达到600万亩。市、县、乡三级农村产权交易平台正式运营，完成土地流转100万亩。重点抓好深化农村集体资产股份制改革试点工作。扎实推进国有林场、农牧场和供销合作社综合改革，加快组建供销集团。积极组建承德农村合作银行。构建新型农业经营体系，培育家庭农场、社区农场200家，农民专业合作社达到10000家。深化国有资产管理改革。健全国控集团运营机制，制定出台国控集团和下属企业薪酬、考核奖惩、资产整合、投资管理等制度，构建融资平台和产业平台互动的资本运营机制，完成首批30亿元债券发行。制定市属32家破产改制企业推进方案，加快推动国有企业改革。深化投融资改革。加大金融市场开放力度，充分利用多层次资本市场和债券、票据等各种金融工具筹集建设资金，全年融资100亿元以上。推动与京津的股权交易、产权交易，探索众筹等多种融资方式激活社会资本，推广“政银企户保”模式。推进交通银行、河北银行、张家口银行设立县级分支机构，新增村镇银行3家。制定上市企业奖励政策，推动企业到中小板、创业板、新三板和区域性股权交易市场挂牌，新增上市企业5家以上。深化财税体制改革。全面实施绩效预算管理改革，建立健全财政资金统筹使用机制，研究出台市以下事权支出责任改革意见。落实好税费改革各项政策。健全完善“借、用、管、还”相统一的政府性债务管理机制，切实防范化解财政金融风险。

(五)强力推进生态环境建设。坚持涵养水源、精准治污、科学防控，确保生态环境质量保持京津冀前列。深入开展水环境综合治理。新增造林绿化面积77万亩，治理水土流失500平方公里。加快实施中小河流治理、河堤水毁工程等23项水源涵养项目，确保双峰寺水库年底前建成蓄水。落实水污染防治“国十条”和“省五十条”，实施取水量、水污染排放量双重控制。启动武烈河流域水环境综合整治三年攻坚行动，确保河流Ⅲ类以上水质断面达标率继续保持在85%以上，饮用水水质达标率持续保持100%。大力推进大气污染防治。深入开展“五大攻坚战”，落实结构减排、工程减排、管理减排措施，实施燃煤治理和清洁能源替代利用，加快老旧车辆更新换代，开展机动车辆污染整治，推进大气污染联防联控，完成省下达的PM2.5年度控制目标，确保环境空气质量二级以上天数稳定在68%以上。实施矿山环境综合整治。启动国家绿色矿业发展示范区创建工作，实施“6743”矿山环境治理工程，年内完成损毁山体治理面积12平方公里，压减矿业权 140个，转产转型矿山企业80家，取缔关停资源品位低、经济效益差、矿山生态环境不达标的矿山企业100家，推动矿山环境持续改善。

(六)强力推进精准扶贫精准脱贫。以脱贫攻坚为抓手，统筹“三农”工作，实现农民富、农业强、农村美。精准帮扶保脱贫。按照中央和省脱贫攻坚的有关要求，扎实做好贫困对象精准识别和建档立卡工作，通过发展生产扶持8.6万人，易地搬迁安置34个贫困村4700余人，就地转化为林业或保护区用工10000 人，教育资助6500人，社会保障、医疗救助兜底解决4.5万人，确保年内15.16万贫困人口稳定脱贫，247个建档立卡贫困村“摘帽”。壮大产业稳脱贫。突出龙头支撑，实施“一环六带”产业扶持行动，新增省级以上龙头企业9家，新增和规范提升市级龙头企业100家以上。突出园区支撑，争创省级现代农业示范园区5个，每个县区至少建设2个现代农业示范园区。优化农业种植结构，压减玉米种植55万亩，新增经济林28万亩、食用菌17万吨、马铃薯11万亩、中药材10万亩、设施菜4万亩。突出农产品标准体系和品牌支撑，围绕扩大京津高端市场，推动实现京承农产品质量互认，力争新增中国驰名商标和省级著名商标 5个以上。做美乡村促脱贫。以美丽乡村建设“十二个专项行动”为抓手，重点抓好兴隆燕山峡谷省级片区和围场御路民俗风情、滦平张百湾产业园区等九大市级精品片区，加快推进平泉和滦平两个省级中心村示范县建设，着力打造231个省级重点村和50个市级精品村，促进“乡村美”与“百姓富”的有机统一。

(七)强力推进新型城镇化建设。着力构建“一核三带”市域城镇体系，加快城乡统筹步伐，提升区域综合实力。老城区开展城市整理。启动“五城联创”工程，坚定不移地实施老城区“中疏”战略，加强历史文化名城保护，通过调整产业业态、严控建设标准、均衡公共服务、完善综合交通等措施，进一步压减存量、控制增量、优化布局。加快推进城市环境和建筑整治、老旧小区改造、城市功能疏解等项目建设，实施中心城区24项绿化美化亮化工程，着力提升城市品位。加快南北新城建设。以存量土地盘活、基础设施配套、重点项目落地为突破口，加快南北两个新城开发建设。南部新城全力推进城市基础设施、土地一级开发和高铁商圈整体规划建设，全面启动道路、管网、电力、防洪等百亿元以上基础设施投资建设计划，启动中心城区72公里滦河综合治理和城市带建设，加快上板城220KV、 110KV变电站建设，确保闫营子大桥、后窑大桥和娘娘庙大桥竣工通车。开工建设承德市儿童医院。北部新城加快贾营路、“01单元”路管网等配套工程建设，推动中国红十字总会健康大学、养老基地等项目签约落地，启动双峰寺水库周边区域开发。推进县城扩容提质。坚持以产兴城、以城兴业、产城教融合，以新一轮县城建设攻坚行动为载体，实施一批旧城整修、公共建筑、美化亮化等景观风貌工程，推动县域经济增比进位。扎实推进产城教一体化，围场、丰宁、滦平3个县教体园区建成，承德县、兴隆、平泉、隆化4个县教体园区开工建设。每个县集中支持一个主业突出、发展潜力较大的产业集群，全市谋划启动建设3—5个以产业为龙头的供给侧经济小镇。以34个市级以上重点乡镇为抓手，建设一批生态型、农贸型、旅游型、工矿型特色小城镇，力争城镇化率提高1.5个百分点。加强基础设施建设。强力推进事关全局和长远的重大基础设施建设，确保京沈客专按时序进度推进、承德机场投入运营、南郊热电力争实现双机发电、京通铁路(承德段)电气化改造顺利开工，承平高速争取开工，围御高速、京锡高速、赤曹高速和津承城际前期工作取得实质性进展。确保承赤高速机场连接线、八里庄连接线和承张高速丰宁连接线建成通车，承张高速隆化、双滦、滦平连接线全面开工。续建和新开工干线公路9条296公里。新开工电网项目9个，新建通讯基站2100座。

(八)强力推进利民惠民工程。把保障和改善民生放在更加突出位置，积极回应人民群众的重大关切。近一时期，围绕办一批关系城乡长远发展和人民群众生产生活的好事实事，我们通过公开向社会征集和认真研究，决定今年重点实施“十项民生工程”，切实办好一批惠民利民实事。主要包括：1.全市城镇新增就业4万人，下岗失业人员再就业6000人，农村劳动力转移就业3.8万人。2.市大学生创业园入驻项目40个，市高层次人才创新创业园入驻项目20个，为优秀人才提供更多的创新创业空间。3.规划实施薄弱学校改造项目303个，投资1.3亿元新建、维修校舍5万平方米;中心城区新建、改扩建会龙山小学、普宁小学、凤凰御景小学及冯营子幼儿园等13所中小学和幼儿园，方便义务教育阶段学生就近上学。4.加大教育扶贫，对建档立卡贫困户中的中等教育学校学生，每生每年给予3000元补助。5.对新生儿实施35种遗传代谢性疾病免费筛查，筛查率达到100%。6.建成市残疾人康复托养中心，改造建设一批社区残疾人、老年人康复站。7.实现低保线与扶贫线“两线合一”，农村低保标准由现行的2295元提升至2800元。8.实施老旧小区环境综合整治，中心城区续建、新增改造项目26个，改造面积58万平方米，使老旧小区脏、乱、差问题得到明显改观。9.启动实施中心城区部分道路及管网建设、整修工程，新建中心城区二仙居旱河南路、开发区东区中路等10条城市道路及管网;对牛圈子沟路道路及管网进行大修;整修武烈路等10条破损严重的城市主次干道道路及管网，完好率达到95% 以上;对大石庙中心街、双峰寺主街等5个城乡结合部实施整治。10.对中心城区佟山中路、佟王府路等9条山地步行系统进行改造，逐步完善武烈河复式河床绿廊步行系统，为市民休闲健身提供更好环境。11.启动实施武烈河市区段16个排污口综合整治，加快解决城市污水经旱河行洪道雨污并混排入武烈河问题，切实改善武烈河的水质和水环境。12.加大解决遗留问题力度，加快回迁房安置工作;新改造棚户区住房5500套，农村危房2万户。13.优化城市公共交通，开通中心城区旅游公交和承德至围场高速客运快线;年内完成9路、29路等公交线路的延时工作，使群众出行更加便捷。14.加强中心城区交通综合整治，新增和配建停车位5700个以上，有效缓解停车难问题。15.实施老城区公厕“旱改水”，年内完成50%以上的改造任务。16.制定实施老旧住宅小区物业管理办法，分步解决老旧小区无物业管理问题;启动小区非供电公司产权配电设施的改造提升，有效解决社区配电设施管护及居民电力供应问题。17.对老城区2.8万米供热管网进行更新改造，消除安全隐患，提高供热质量。18.对窝瓜园等污水管道进行改造，铺设大石庙东山道路、汇水湾B区道路地下管网。19.实施有线电视标清改高清工程，提高承德电视台3个频道播出质量。20.严格规范老城区室外商业活动，清理店外占道经营和商业宣传活动噪音扰民,为市民提供健康安宁的生活环境。

各位代表!人民对美好生活的向往就是我们的奋斗目标，保障和改善民生是我们对全市人民的庄严承诺。我们一定时刻把人民的安危冷暖放在心上，时刻把人民的嘱托期待记在心头，下大力保障和改善民生，抓好安全生产、食品药品安全及森林草原防火等各项安全工作，深入实施“七五”普法，严厉打击违法犯罪，有效防控各种风险，确保社会大局和谐稳定，让全市人民生活得更有尊严、更加幸福安康!

各位代表!新起点、新征程、新使命，尽管前进的路上还有很多困难、风险和挑战，但我们将坚持依法行政、廉洁从政、从严治政，全面加强政府自身建设，为实现各项目标任务提供坚强保障。着力建设改革创新的政府，进一步推进解放思想大讨论，打破因循守旧的惯性思维，敢于创新突破，增强战略眼光，在探索中寻找路径、在尝试中积累经验、在实践中争创一流。着力建设依法履职的政府，遵守宪法和法律，坚持法律底线不可逾越，法定职责必须为、法无授权不可为，推进行政权力公开，强化行政权力监督，规范行政权力运行，自觉接受人大法律监督和工作监督、政协民主监督和社会监督，让政府的每一项重大决策都经得起历史和人民的检验。着力建设勤政务实的政府，牢固树立功成不必在我的理念，始终把维护和实现人民利益放在至高无上的位置，多干打基础、利长远、惠民生的好事实事。大力弘扬敬业奉献精神、创先争优精神和团结协作精神，夙兴夜寐、激情工作、久久为功，建立健全推动落实的责任机制、奖罚分明的激励问责机制，以过硬的作风确保工作实效。着力建设担当负责的政府，大力弘扬抓铁有痕、踏石留印和钉钉子精神，坚持“干”字当头，强化激励约束，建立完善政府服务企业直通车机制，探索实行鼓励担当负责的容错免责机制，推动形成面对矛盾和困难不回避、敢碰硬、肯担当，层层尽责促改革、人人作为谋发展的崭新局面。着力建设廉洁高效的政府，把纪律和规矩挺在前面，恪守《准则》、《条例》，严守“八项规定”，强化“两个责任”，践行“三严三实”，坚决整治不作为、慢作为、乱作为，切实提高行政效能，为加快承德改革发展创造更优的环境，为基层、企业和广大人民群众提供更好的服务，以实实在在的工作业绩向省委、省政府，向全市人民交一份满意的答卷!

各位代表!“十三五”描绘的宏伟蓝图令人鼓舞，全市人民对美好生活的追求和向往催人奋进。让我们更加紧密地团结在以习近平同志为总书记的党中央周围，在省委、省政府和中共承德市委的坚强领导下，高举发展、团结、奋斗的旗帜，弘扬正气、凝心聚力、真抓实干、奋勇争先，为全面建成小康社会，加快建设“生态强市、魅力承德”而努力奋斗!
